# Dynamic changes of serum protein in rats with acute intoxication of Chinese cobra snake venom by proteomic analysis

**DOI:** 10.1080/20961790.2017.1405565

**Published:** 2017-12-21

**Authors:** Hui Yan, Ping Xiang, Jingshuo Zhang, Liqi Xie, Min Shen

**Affiliations:** a Shanghai Key Laboratory of Forensic Science, Shanghai Forensic Platform, Department of Forensic Toxicology, Academy of Forensic Science, Shanghai, China; b College of Pharmaceutical Sciences, Soochow Universtity, Suzhou, Jiangsu, China; c Institutes of Biomedical Sciences, Fudan University, Shanghai, China

**Keywords:** Forensic sciences, forensic toxicology, proteomics, snake bite, cobra, serum, rats

## Abstract

To elucidate the toxic mechanism of snake venom at the protein level, proteomics technology was applied to investigate the effect of venom on circulation in the mammalian body. Temporal proteomic analysis was performed to profile the dynamic changes in the sera of Sprague–Dawley rats administered with Chinese cobra venom or saline. Using 8-plex iTRAQ analysis, 392 and 636 serum proteins were identified to be linearly upregulated or downregulated over time in the low-dose group and high-dose group, respectively. These proteins were mainly associated with the acute phase response pathway, complement system, and liver X receptor (LXR)/retinoid X receptor (RXR) and farnesoid X receptor (FXR)/RXR activation pathways. Compared with the low-dose group, the immune response and integrin pathways were inhibited in the high-dose group, although no obvious effect was observed. With consistently higher or lower expression in the high-dose group compared to the low-dose group throughout the whole process of venom poisoning, two proteins, Kininogen-1 (KNG1) and orosomucoid 1 (ORM1), which are involved in metabolism and immune response, occupied a core position in the pathway network and are considered venom dose-dependent biomarker candidates.

## Introduction

Venomous snakebite is a frequently devastating albeit neglected public health threat [1,2]. There are more than 2 700 species of snakes worldwide, and about one-fifth of them are venomous and are capable of producing venom with biological activities from venom glands, favouring the capture and digestion of prey [3]. Once injected into the bloodstream, snake venom spreads out into the body through circulation and causes various local or systematic poisoned symptoms. Approximately 100 000 snakebites occur annually in China, of which 73% of victims are young and middle-aged. Approximately 5%–10% of the victims were killed by snakebite, and another 25%–30% of the victims suffer from permanent tissue damage [4]. The Chinese cobra (*Naja atra*) is a member of the Elapidae family and one of the 10 most venomous snakes in China [5]. The effects of the Chinese cobra bite are acute and critical, and it is commonly observed in emergency departments. The Chinese cobra can spit large amounts of venom containing both neurotoxin and haemotoxin. Highly lethal neurotoxins may result in neuromuscular inhibition, muscle paralysis and respiratory failure, while haemotoxins can destroy red blood cells, causing a breakdown or inflammation in the body. Rapid absorption of the neurotoxin and haemotoxin from the injection site into the systemic circulation indicates the fast onset of these principal toxins, which are responsible for the early systemic manifestation of envenoming [6].

In the early days, the mechanism of venom toxicity was generally studied using traditional methods, such as immunofluorescence, ELISA and enzyme activity detection [7–9]. However, these methods exhibit low efficiency and are more likely to miss key factors. As an emerging technology, mass-spectrometry-based proteomics has been widely employed in the study of protein profiles and proprieties in body fluids, including serum, cerebrospinal fluid, nipple leaching fluid, tears, saliva, amniotic fluid, urine and ascites [10]. Proteomic tools have been used to study the composition and cross-reactivity of anti-venoms with venoms to address the pathology of snakebite envenoming [11]. Malih et al. [12] elucidated the venom proteome of *Naja haje legionis* using a combination of size exclusion chromatography, reverse-phase high-performance liquid chromatography (HPLC), Tricine/SDS-Page and Q-TOF tandem mass spectrometry. *N*. *haje legionis* venom contained neurotoxic activities, inducing irreversible blockade of neuromuscular transmission in both rodent and chick nerve-muscle preparations, and exhibited myotoxic and cardiotoxic activities [13]. In addition, a functional proteomic approach was used to identify geographic variations of king cobra venoms from southeast Asia and China [14]. Thus, proteomics may serve as a powerful tool to study the mechanism of venom toxicity on a global scale.

Snake venoms were analyzed by “venomics”, a proteomic strategy used to determine their composition [15–17]. Venoms of the *Naja* species can induce a predominantly cytotoxic pattern of envenoming that may evolve into tissue necrosis and gangrene [18]. Tanaka et al. [19] found that the venom of *Micrurus nigrocinctus*, a species of venomous elapine snake, could activate the complement pathway. Once activated, the complement system produces a large number of anaphylatoxin to promote vasodilation and thus accelerate the spread of other toxic proteins in circulation. An *L*-amino acid oxidase from *Ophiophagus hannah* snake venom demonstrated potent inhibitory activity on platelet aggregation induced by ADP and U46619 [20]. However, few systemic studies have been performed about serum proteins after intoxication of Chinese cobra venom.

Therefore, a proteomics approach was performed to systematically study the effect of snakebite time and venom dose on serum proteins, in order to investigate the alteration of serum proteins caused by Chinese cobra venom. First, Sprague–Dawley (SD) rats were used to establish the snakebite mode, and dose tests were performed to determine the lethal dose. Next, rat sera 1, 2 and 4 h post-poisoned were collected and analyzed using proteomics to understand the process of venom entrance into the circulation. By analyzing the proteome alteration in the haematological system after venom entrance, it may provide additional insight on the effect of venom on circulation and identify potential biomarkers for intoxication of Chinese cobra venom, which may be useful in forensic toxicology.

## Materials and methods

### Instruments and reagents

Chinese cobra venom was obtained from a rainbow snake farm in Yingtan, China. All reagents used in this study were purchased from Sigma (St. Louis, MO, USA) unless specified otherwise. Isobaric tags for the relative and absolute quantification kit was purchased from AB Sciex (Framingham, MA, USA). Acetonitrile hypergrade was obtained from Merck (Darmstadt, Germany). The ProteoMiner protein enrichment kit was purchased from BIO-RAD (Hercules, CA, USA). The 2D Clean-up kit and Plus One 2-D Quant kit were purchased from GE Healthcare (Uppsala, Sweden).

### Rat models of snakebite

SD rats (450–600 g) of SPF grade were obtained from Shanghai SLAC Laboratory Animal Co., Ltd. Lyophilized venom powder from Chinese cobra were dissolved in saline in a concentration series and intramuscularly injected into the rat leg, respectively. The lethal dose was selected as the amount that results in death 4 h post-injection, which was 0.04 mg venom per 100 g rat weight.

Rats were divided into three groups according to the dosage as follows: the high-dose group (lethal dose, 0.4 µg/g, *n* = 12), low-dose group (half the lethal dose, 0.2 µg/g, *n* = 8) and control group injected with 0.2 mL saline (*n* = 6). Sera were collected from the rat's orbit at 1, 2 and 4 h post-injection. After 4 h of injection, all of the surviving rats were sacrificed by cervical dislocation.

### Enrichment of low-abundance serum proteins and iTRAQ labelling

Low-abundance serum proteins were concentrated using the ProteoMiner enrichment kit according to the manufacturer's instructions and further purified using the 2D Clean-up kit. Precipitated proteins were re-dissolved in the solution provided in the iTRAQ kit and the concentration was determined using the PlusOne 2-D Quant kit. Next, 100 µg protein were used for iTRAQ labelling proteomics analysis. Protein digestion and labelling were performed according to the manufacturer's instructions. Briefly, after reduction and alkylation, proteins were digested for 16 h at 37 °C using sequencing grade modified trypsin (*W*_protein_:*W*_enzyme_ = 50:1). Digested peptides from each sample were then labelled with different iTRAQ tags and incubated at room temperature for 1 h. Labelled peptides were then mixed, dried under vacuum and stored at −20 °C for subsequent analysis.

### 2D-LC/MS analysis

iTRAQ labelled peptides were re-dissolved in 50 µL buffer A (10 mmol/L KH_2_PO_4_, 25% acetonitrile, pH = 2.6) and separated on a Polysulfoethyl SCX column (2.1 mm × 100 mm, 5 µm, 200 Å) offline using Shimadzu Liquid chromatography consisting of binary pumps LC20-AD (Shimadzu, Kyoto, Japan) with UV detection operated at 214 nm/280 nm. Samples were automatically loaded onto the column and eluted with a linear 60-min gradient of 5%–25% solvent B (10 mmol/L KH_2_PO_4_, 350 mmol/L KCl, 25% acetonitrile, pH = 2.6) at a flow rate of 200 nL/min. The 20 collected fractions were desalted and dried under vacuum for subsequent analysis.

Peptide fractions obtained from SCX were reconstituted in 50 µL buffer A (50% ACN, 0.1% formic acid) and separated on a ZORBAX 300SB-C18 column (0.1 mm × 15 mm, 5 µm, 300 Å). Reverse phase high-performance liquid chromatography was performed using the Shimadzu LC20-AD system. Peptides were loaded onto a trap column at 60 µL/min and eluted with a 90-min gradient of 5%–35% solvent B (95% ACN, 0.1% formic acid) at 300 nL/min. MS data were acquired using the data-dependent proteomics method on the Q-Exactive mass spectrometer (ThermoFisher Scientific, San Jose, CA, USA) equipped with a HESI-II Spray source. Nitrogen was used as both the sheath gas and the auxiliary gas at 35 bar and 10 bar, respectively. HESI source parameters were set as capillary temperature 320 °C, spray voltage of 3 800 V and heater temperature 300 °C. In each cycle, a full scan was acquired with a mass range of *m*/*z* 400–1 800. The most intense precursor ions were selected and subsequently fragmented by higher energy collisional dissociation (HCD).

### Data analysis

The raw MS data from data-dependent acquisition (DDA) were analyzed by Peaks Studio (Thermo Fisher) for protein identification and quantification. K-means clustering and Pearson correlation coefficient analysis were performed to identify proteins with linearly increased or decreased expression over time in each of the poisoned groups (low and high dose). These proteins were further analyzed using the Ingenuity Pathway Analysis platform (IPA, QIAGEN, Redwood City, CA, USA; www.qiagen.com/ingenuity), including downstream effect analysis, pathway analysis, regulator effect analysis and protein–protein interaction analysis, in order to investigate the corresponding biological response over the time course. Subsequently, proteins in each poisoned group were further compared with the control group, and those with a ratio less than 0.7 or greater than 1.33 were defined as significant proteins [21].

## Results and discussion

### Behavioral changes of rats after the injection of Chinese cobra venom

Immediately after injection with a lethal dose of Chinese cobra venom, the rats’ activities were attenuated and their injected limb appeared to be paralyzed. The injected limb swelled 30 min later. One hour post-injection, the rats’ lips became purple, and their eyes appeared to be dark and some of the rats had rales. Two hours later, the rats began to twitch and die. Four hours after injection, the rats that were still alive were sacrificed and the injected sites and organs were harvested for observation. A bruise was observed in the muscle and skin at the injected site. The lungs were darker than the control group, while the other organs were normal.

After injection with a low dose of Chinese cobra venom, the poisoned symptoms were similar as those described above, which are declined physical activities, paralyzed limbs, purple lips, dark eyes and rales. However, all of the poisoned rats still survived 4 h after injection. Similarly, a bruise was also observed in the muscle at the injected site and the lungs also appeared darker than the control group, while the other organs looked normal.

### Time course study of serum proteome profiles after venom injection

**Figure 1.**
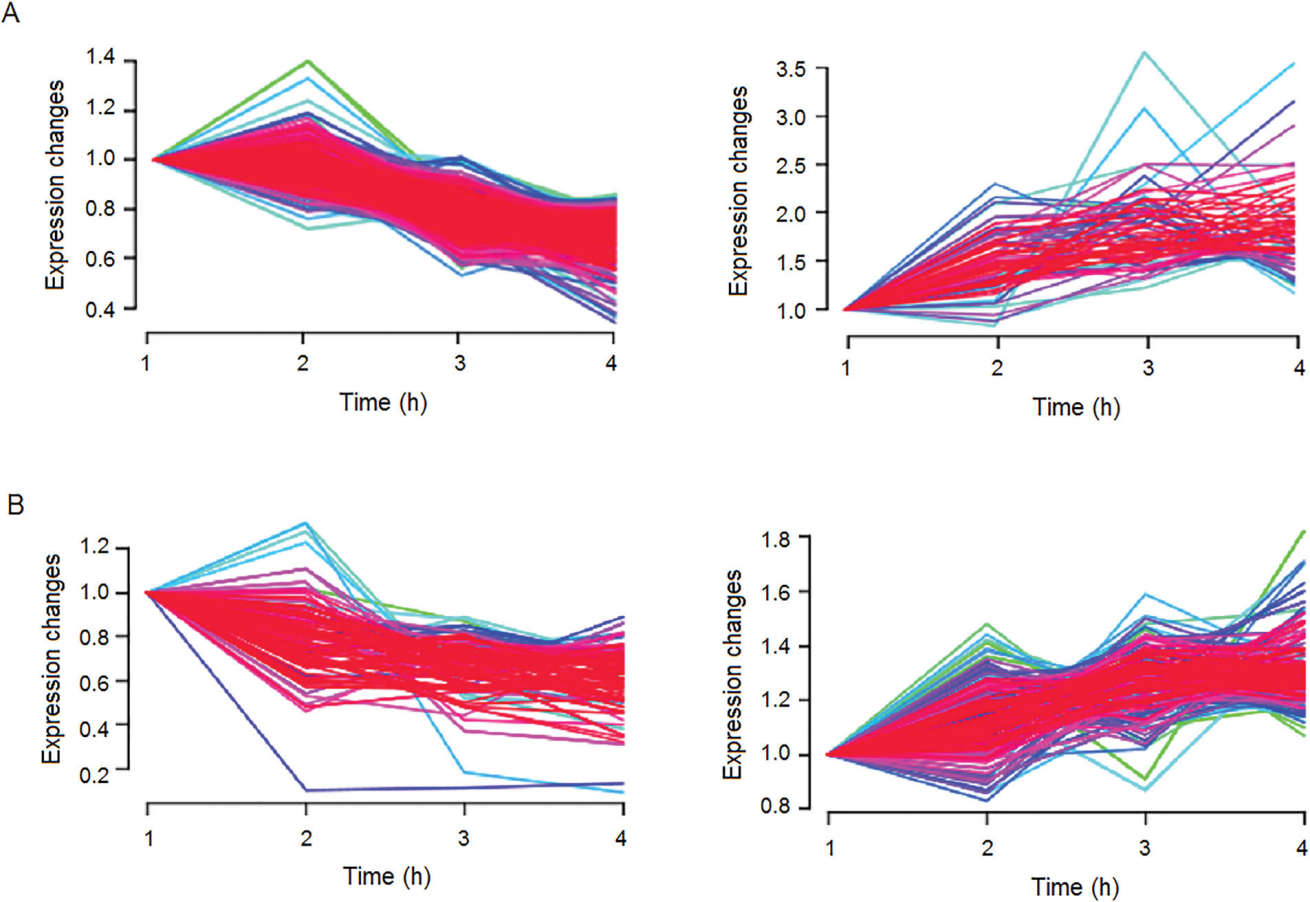
Cluster analysis of proteins persistently decreased or increased over the observed time points for the high-dose group (A) and low-dose group (B).

To investigate the effect of venom on serum proteins, iTRAQ was employed to compare the serum proteome change between the control group and poisoned rats (high-dose group and low-dose group) 1, 2 and 4 h post-venom injection. A total of 1 565 proteins were identified, and 367 of these were venom proteins. Serum proteins linearly upregulated or downregulated over time could serve as potential biomarkers to indicate the effect of venom on circulation. In this study, proteins persistently increased or decreased over the observed time points (0, 1, 2 and 4 h) and with absolute number of Pearson correlation coefficient over 0.8, *P*-value < 0.05 were taken as candidates (Figure 1). There were 392 dysregulated proteins in the low-dose group and 636 in the high-dose group. Interestingly, the expressions of most candidates were linearly decreased in either the high- or low-dose group, indicating that venom entrance could result in severe damage to the rat's circulation.

### Significantly regulated proteins in serum after venom entrance

The expression of serum proteins in the dosage group was further compared with the control group and proteins with a ratio of less than 0.7 or greater than 1.33 were determined as proteins with significantly altered expression (Tables 1 and 2).

**Table 1.** Significantly changed serum proteins in low-dose group.

Accession	Description	1 h	2 h	4 h	MW
P10719	ATP synthase subunit β, mitochondrial	0.565	0.520	0.576	56 300
P10960	Sulfated glycoprotein 1	0.583	0.528	0.639	61 100
P21533	60S ribosomal protein L6	0.607	0.686	0.556	33 500
Q6P6S4	Nucleotide exchange factor SIL1	0.607	0.614	0.663	52 300
Q62975	Protein Z-dependent protease inhibitor	0.607	0.454	0.556	50 200
P18418	Calreticulin	0.648	0.459	0.635	48 000
Q64537	Calpain small subunit 1	0.654	0.558	0.578	28 600
Q8CGX0	Insulin-like growth factor 2 mRNA-binding protein 1	0.654	0.354	0.373	63 400
Q5XIM9	T-complex protein 1 subunit β	0.684	0.621	0.483	57 400
Q8K3U6	Coagulation factor VII	0.686	0.605	0.671	50 400
P69897	Tubulin β-5 chain	0.689	0.404	0.395	49 600
P60571	Pannexin-2	1.359	1.551	1.633	73 200
Q6AXZ4	Uncharacterized protein C6orf182 homolog	1.360	0.493	0.415	50 200
P02091	Haemoglobin subunit β-1	1.376	1.607	1.406	16 000
P00762	Anionic trypsin-1	1.394	1.626	1.386	25 900
P23680	Serum amyloid P-component	1.403	1.644	1.330	26 200
P13803	Electron transfer flavoprotein subunit α, mitochondrial	1.432	1.993	1.835	34 900
P39069	Adenylate kinase isoenzyme 1	1.443	1.379	1.383	21 600
Q5RJZ6	Short coiled-coil protein	1.511	3.407	3.010	13 700
P04466	Myosin regulatory light chain 2, skeletal muscle isoform	1.581	1.743	2.639	19 000
P01946	Haemoglobin subunit α-1/2	1.600	1.729	1.734	15 300
P11517	Haemoglobin subunit β-2	1.620	1.649	1.403	16 000
P30836	L-selectin	1.732	1.618	1.561	42 400
P09739	Troponin T, fast skeletal muscle	1.770	3.061	3.655	30 700
Q64428	Trifunctional enzyme subunit α, mitochondrial	1.905	1.836	1.573	82 600
P13413	Troponin I, slow skeletal muscle	2.241	2.326	2.065	21 700
Q66H15	Regulator of microtubule dynamics protein 3	2.291	0.672	0.434	52 300
P19234	NADH dehydrogenase [ubiquinone] flavoprotein 2, mitochondrial	3.524	4.613	5.997	27 400

The expression of serum proteins in the dosage group was further compared with the control group and proteins with a ratio of less than 0.7 or greater than 1.33 were determined as proteins with significantly altered expression. MW: molecular weight.

**Table 2.** Significantly changed serum proteins in high-dose group.

Accession	Description	1 h	2 h	4 h	MW
Q6AXZ4	Uncharacterized protein C6orf182 homolog	0.387	0.429	0.511	50 200
P04916	Retinol-binding protein 4	0.425	0.403	0.301	23 200
Q62975	Protein Z-dependent protease inhibitor	0.459	0.488	0.509	50 200
P08494	Matrix Gla protein	0.470	0.615	0.397	12 000
P69897	Tubulin β-5 chain	0.497	0.490	0.490	49 600
P53813	Vitamin K-dependent protein S	0.507	0.531	0.535	74 600
P10960	Sulfated glycoprotein 1	0.547	0.609	0.600	61 100
Q6AYG3	Protein prune homolog	0.560	0.496	0.575	50 000
P40307	Proteasome subunit β type-2	0.566	0.564	0.486	22 900
P38659	Protein disulfide-isomerase A4	0.571	0.598	0.658	72 700
O88269	Multidrug resistance-associated protein 6	0.575	0.599	0.660	164 900
P62828	GTP-binding nuclear protein Ran	0.577	0.450	0.400	24 400
Q5M889	Apolipoprotein F	0.600	0.697	0.593	33 800
P18418	Calreticulin	0.600	0.540	0.655	48 000
Q9QZA2	Programmed cell death 6-interacting protein	0.610	0.543	0.605	96 600
P40112	Proteasome subunit β type-3	0.639	0.634	0.624	22 900
P22062	Protein-L-isoaspartate(D-aspartate) O-methyltransferase	0.676	0.592	0.641	24 600
Q9R1T1	Barrier-to-autointegration factor	0.678	0.595	0.416	10 000
Q5RK00	39S ribosomal protein L46, mitochondrial	0.688	0.633	0.306	31 700
P29534	Vascular cell adhesion protein 1	1.338	1.571	1.393	81 200
P01946	Haemoglobin subunit α-1/2	1.354	1.508	2.622	15 300
Q62826	Heterogeneous nuclear ribonucleoprotein M	1.410	1.424	1.474	73 700
P17764	Acetyl-CoA acetyltransferase, mitochondrial	1.422	1.591	1.697	44 700
P62989	Ubiquitin	1.423	2.482	3.492	8 600
Q7TNY6	Golgi resident protein GCP60	1.534	1.387	1.634	60 400
Q9Z0W0	Glucagon-like peptide 2 receptor	1.554	1.640	1.344	63 100
P23680	Serum amyloid P-component	1.560	1.693	1.484	26 200
Q62725	Nuclear transcription factor Y subunit gamma	1.659	1.738	1.407	37 200
P04906	Glutathione S-transferase P	1.702	1.479	1.638	23 400
Q9QUH3	Apolipoprotein A-V	1.710	1.737	1.361	41 400
Q80Z29	Nicotinamide phosphoribosyltransferase	1.735	1.578	1.473	55 400
A4L9P7	Sister chromatid cohesion protein PDS5 homolog A	1.749	1.784	2.232	150 200
O88941	Mannosyl-oligosaccharide glucosidase	1.786	1.993	1.617	91 800
Q62818	Translation initiation factor eIF-2B subunit β	1.790	1.585	1.512	38 900
Q66H15	Regulator of microtubule dynamics protein 3	1.824	0.606	0.612	52 300
P60571	Pannexin-2	1.951	1.659	1.857	73 200
P11915	Non-specific lipid-transfer protein	1.984	1.792	2.295	58 800
P13803	Electron transfer flavoprotein subunit α, mitochondrial	2.196	2.994	1.844	34 900
P30836	L-selectin	2.254	1.788	1.687	42 400
O35870	Fanconi anaemia group C protein homolog	2.300	2.300	5.781	63 600
Q5M9G7	Digestive organ expansion factor homolog	2.466	2.428	2.251	87 800
Q641Y6	Protein phosphatase 1J	2.597	1.902	2.141	55 200
P13413	Troponin I	3.090	3.427	2.720	21 700
Q5RJZ6	Short coiled-coil protein	3.392	3.969	2.752	13 700
Q64428	Trifunctional enzyme subunit α, mitochondrial	3.449	2.514	2.815	82 600
P19234	NADH dehydrogenase [ubiquinone] flavoprotein 2, mitochondrial	3.597	4.447	5.986	27 400
P97536	Cullin-associated NEDD8-dissociated protein 1	4.776	2.278	1.479	136 300

The expression of serum proteins in the dosage group was further compared with the control group and proteins with a ratio of less than 0.7 or greater than 1.33 were determined as proteins with significantly altered expression. MW: molecular weight.

Most of the significantly altered serum proteins were identified in both the high- and low-dose groups, with a similar upregulation or downregulation profile. For example, the consistent upregulation of haemoglobin indicated that cobra venom caused damage to red cells and such an observation was consistent with the bruise observed at the injected sites. The upregulation of Tropon indicated a heart problem after injection. Pannexin-2 predominantly exists as transmembrane channels and is primarily expressed in the nervous system, predominantly in the brain and spinal cord [22]. The persistent increased expression of Pannein-2 in rat serum suggested that cobra venom could destroy neurons and release Pannexin-2 into the blood. Tubulin is a major component of microtubules and predominantly exists in the cytoplasm and cytoskeleton [23]. Downregulation of tubulin post-venom injection indicated that cobra venom could destroy tubulin protein, subsequently disrupting cells. Reports have shown that matrix metalloproteinase is upregulated after the destruction of tubulin, and thus promotes the digestion of collagen and proteoglycan and increases cell apoptosis in the early stage. Taken together, these findings indicate neural and hematic toxicities in cobra venom.

Some significantly altered proteins, such as anionic trypsin, 60S ribosomal protein L6, adenylate kinase isoenzyme 1, coagulation factor VII, insulin-like growth factor 2 mRNA-binding protein 1, calpain small subunit 1, T-complex protein 1 subunit β, ATP synthase subunit β, troponin and nucleotide exchange factor SIL1, were unique for the low-dose group (Table 1). The upregulation of anionic trypsin suggested that Chinese cobra venom could cause damage to the pancreas, as anionic trypsin was also upregulated in the serum of patients with pancreatic carcinoma [24]. 60S ribosomal protein L6, a ribosomal protein, was downregulated in rat serum after venom injection. This protein could be decreased during neuronal differentiation [25]. Chinese cobra venom contains nerve growth factor (NGF), which is involved primarily in the growth, proliferation and survival of neurons. Thus, this finding suggested that neuronal differentiation in rats could be affected upon venom entrance. Adenylate kinase isoenzyme 1 is an adenylate kinase, which is upregulated during neuronal differentiation [26]. Its increased expression could be associated with the NGF in venom. Calreticulin is a Ca^2+^-dependent protein that is widely distributed in eukaryotic cells. Once calreticulin binds with Ca^2+^, it is activated and can trigger an array of kinases and phosphatases, thereby regulating the metabolism. As an important protein involved in signalling transduction, calreticulin exerts multiple biological functions, such as inflammation, metabolism and cell apoptosis, in which many critical enzymes are involved [27–29]. In this study, cobra venom injection resulted in the downregulation of calreticulin, which reduces the binding of Ca^2+^ ions and renders calreticulin inactive. Thus, various biochemical reactions in rats were hindered, which further resulted in the multiple poisoned symptoms.

Some proteins that could be associated with the toxicity mechanism of venom were found in the high-dose venom group (Table 2). Retinol-binding protein 4 (RBP4), a low molecular protein, belongs to the lipocalin family and is the specific carrier for retinol in blood from the liver to the peripheral tissues. This protein can specifically bind to retinal pigment epithelial cells and provide retinol. RBP4 is widely distributed in blood, urine and other body fluids. Due to its low molecular weight and short half-life, RBP4 is important for clinical diagnosis and prognosis of liver and renal disease. Several studies [30–32] have shown that its serum concentration could be decreased during liver injury or suffered with dysfunction of renal tubular reabsorption. In our study, RBP4 was linearly downregulated over time, indicating that cobra venom could cause acute injury to rat liver and renal systems. The acute injury of the renal system by the venom has been confirmed in previous studies [33,34], and there has been no literature referring to the hepatotoxicity of venom. Because venom has shown its power in the treatment of hepatic fibrosis, it is possible to affect live function, and further study is needed. RBP4 may be the potential biomarker of cobra venom toxicosis. In the high-dose group, we found some significantly altered proteins associated with mitochondria, such as electron transfer flavoprotein, acetyl-CoA acetyltransferase, NADH dehydrogenase [ubiquinone] flavoprotein 2, 39S ribosomal protein L46 and trifunctional enzyme. These results implied that cobra venom could damage the function of mitochondria. Mitochondria are the primary energy-providing organelles, and its damage may result in various abnormal symptoms [35,36].

### Biological alteration of serum after venom entrance

**Figure 2.**
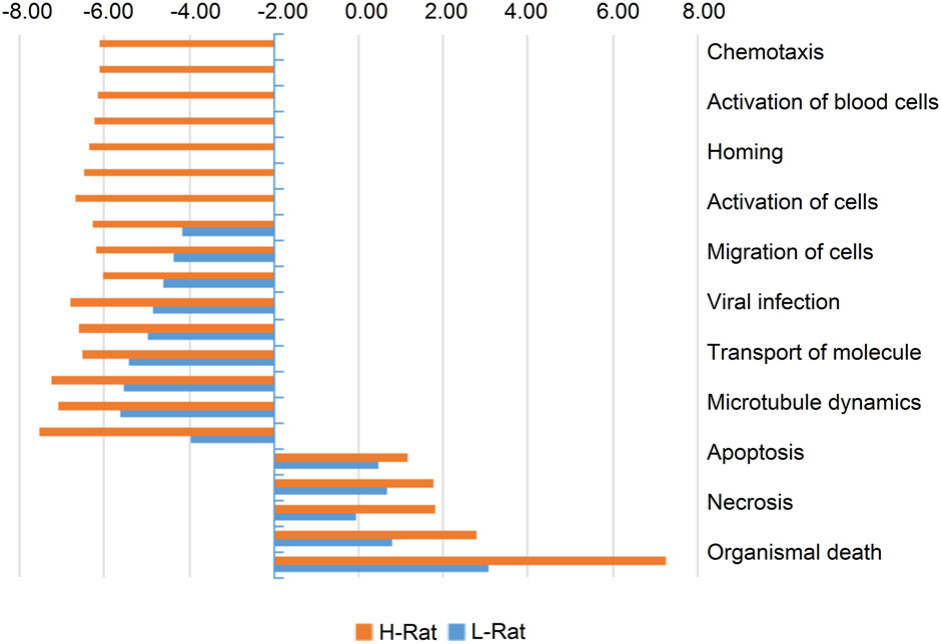
Differential biological effects between the high- and low-dose groups. The negative value indicates an inhibited biological effect, while the positive value indicates activation.

**Figure 3.**
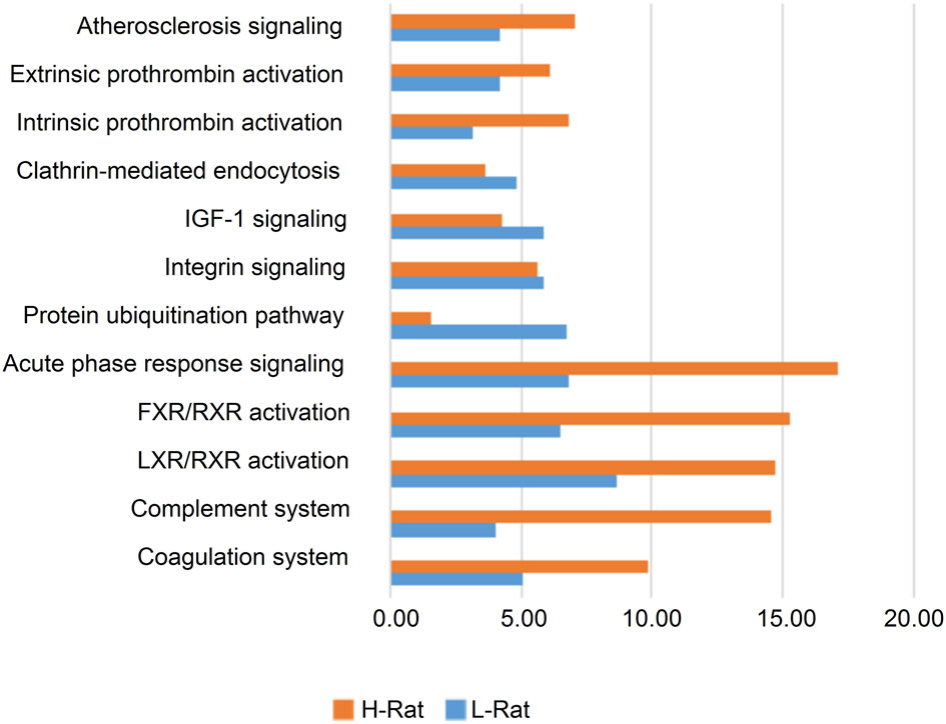
Dysregulated pathways. The *x*-axis represents a significant score, −log(*P*-value). The larger the value, the more significant the dysregulation.

**Figure 4.**
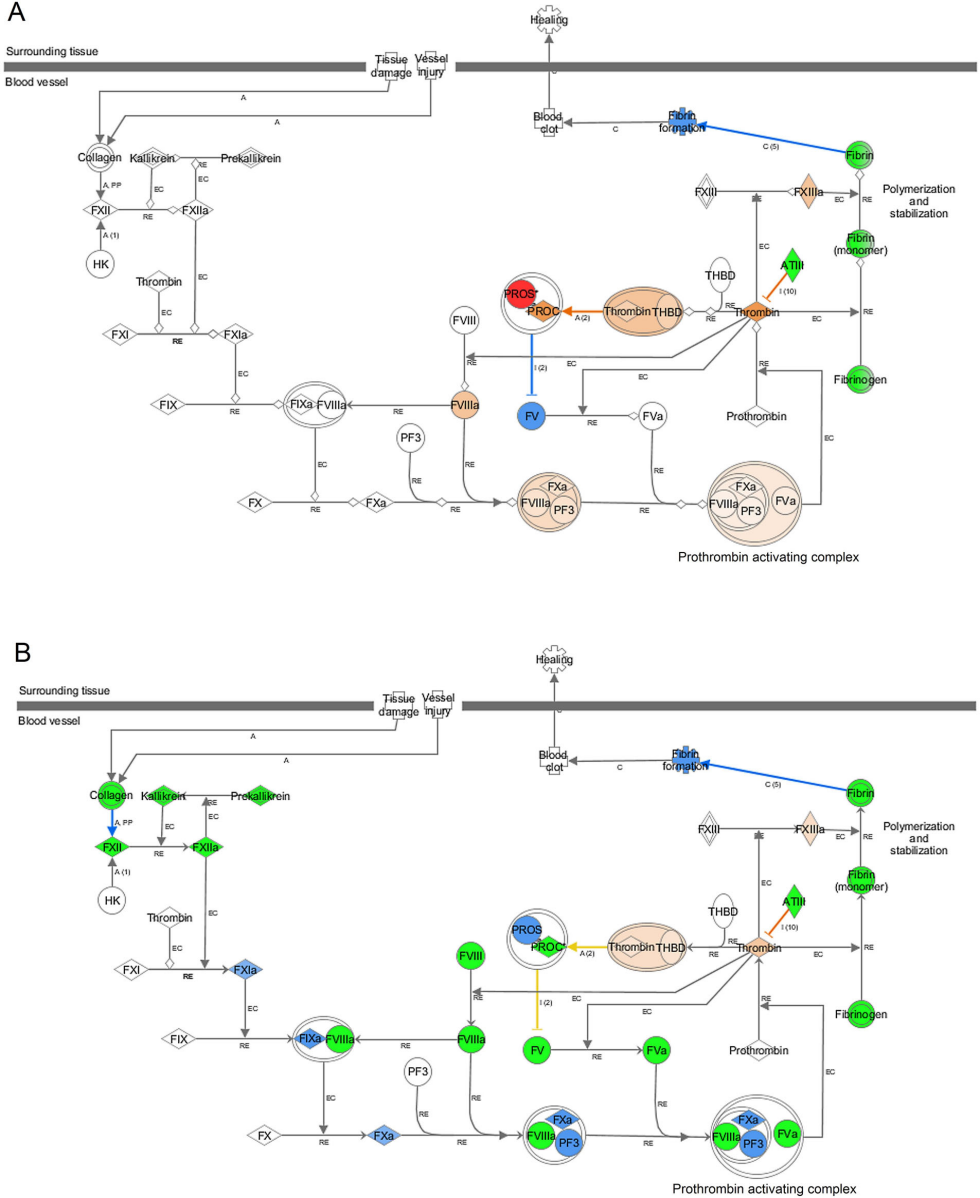
The prothrombin activation pathway: (A) low dose; (B) high dose. Proteins in red and green represent upregulation and downregulation, respectively. Blue and yellow represent proteins predicted to be inhibited and activated, respectively. The darker the colour, the more significant the response.

**Figure 5.**
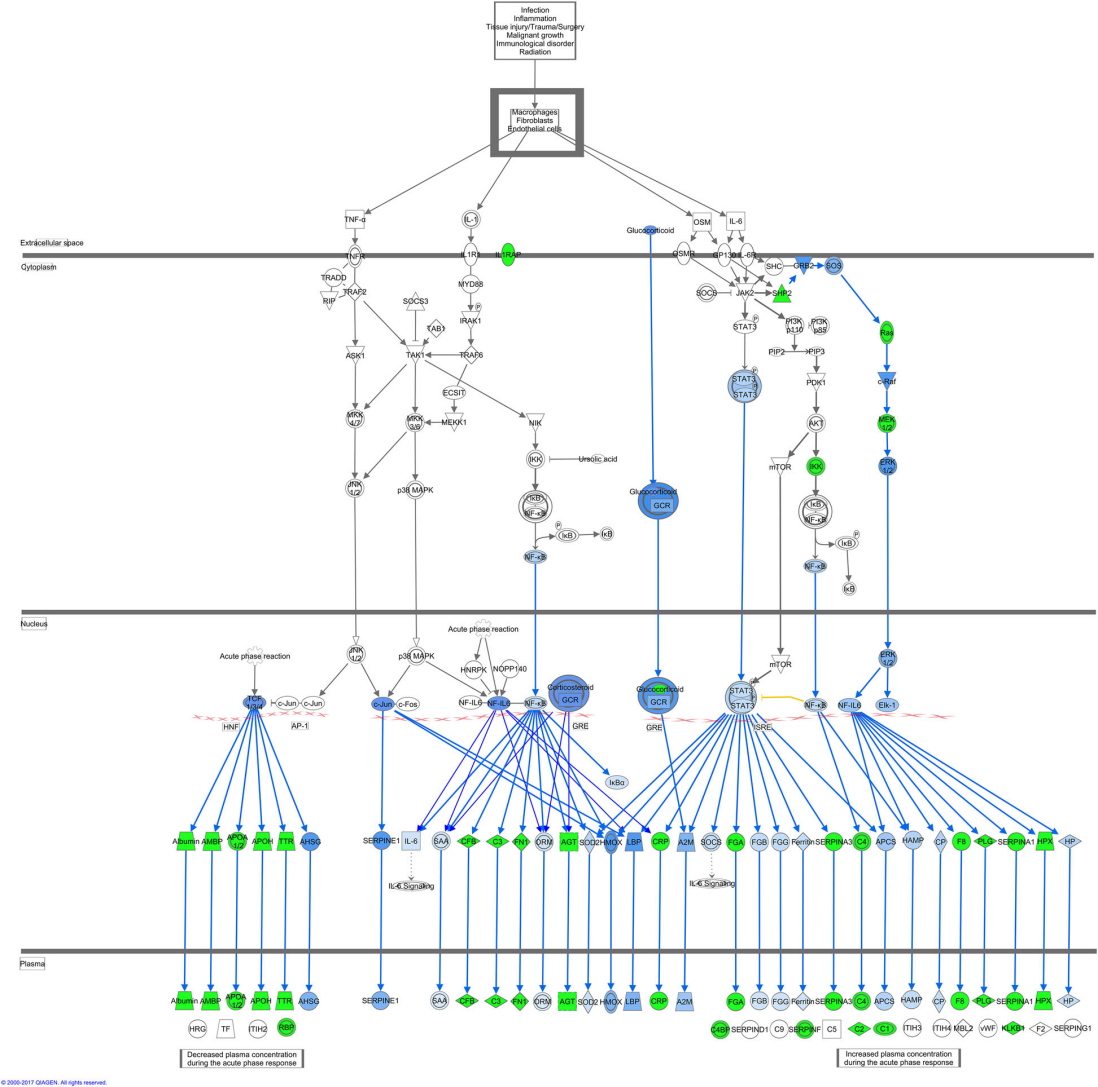
The acute phase response signalling pathway in the high-dose group (this pathway could help the host to defend itself against an infection and trauma via acute immunological regulation). In the high-dose group, most of the proteins in this pathway were downregulated (green) or predicted to be inhibited (blue), indicating that such a response was inhibited by a high dose of venom entering.

These candidates were further examined using IPA proteome software to investigate the corresponding biological responses over time, including downstream effect analysis, pathway analysis, regulator effect analysis and protein–protein interaction analysis. First, downstream effect analysis was performed and those with the most significantly affected functions are displayed (Figure 2). Most of these functions were much more dysregulated in response to the high-dosage group compared to the low-dosage group, indicating its more severe toxicity to circulation. Subsequently, enriched pathway analysis was performed and a comparison was made between the two poisoned groups (Figure 3). As shown in Figure 4, the prothrombin activation pathway was clearly inhibited in the high-dose group, indicating that a higher dose of venom caused severe damage to the rats. However, such a response appeared slightly ambiguous in the low-dose group. As shown in Figure 5, the acute phase response pathway was significantly inhibited in the high-dose group over time, indicating that the acute response system was destroyed and, thus, the rats failed to effectively fight against the venom invasion. Other pathways, such as the complement system, liver X receptor (LXR)/retinoid X receptor (RXR) and farnesoid X receptor (FXR)/RXR activation pathways associated with the immune response and integrin pathway, were also obviously inhibited. In addition, enzymes involved in glycolysis and some metabolic pathways involved in the synthesis of nitric oxide and oxygen were also significantly inhibited in the high-dose group. In contrast, no such effects were observed in the low-dose group.

The regulatory effects feature in IPA empowers generation of a hypothesis for how a phenotype, function or disease is regulated by particular dysregulated proteins by activated or inhibited upstream transcription factors. Consequently, compared to the low-dose venom, we found that most of the upstream transcription factors in the high-dose group were more associated with the dysregulation of cell migration, adhesion and differentiation, and their major effects on biological functions were the inhibition of development, differentiation and migration.

**Figure 6.**
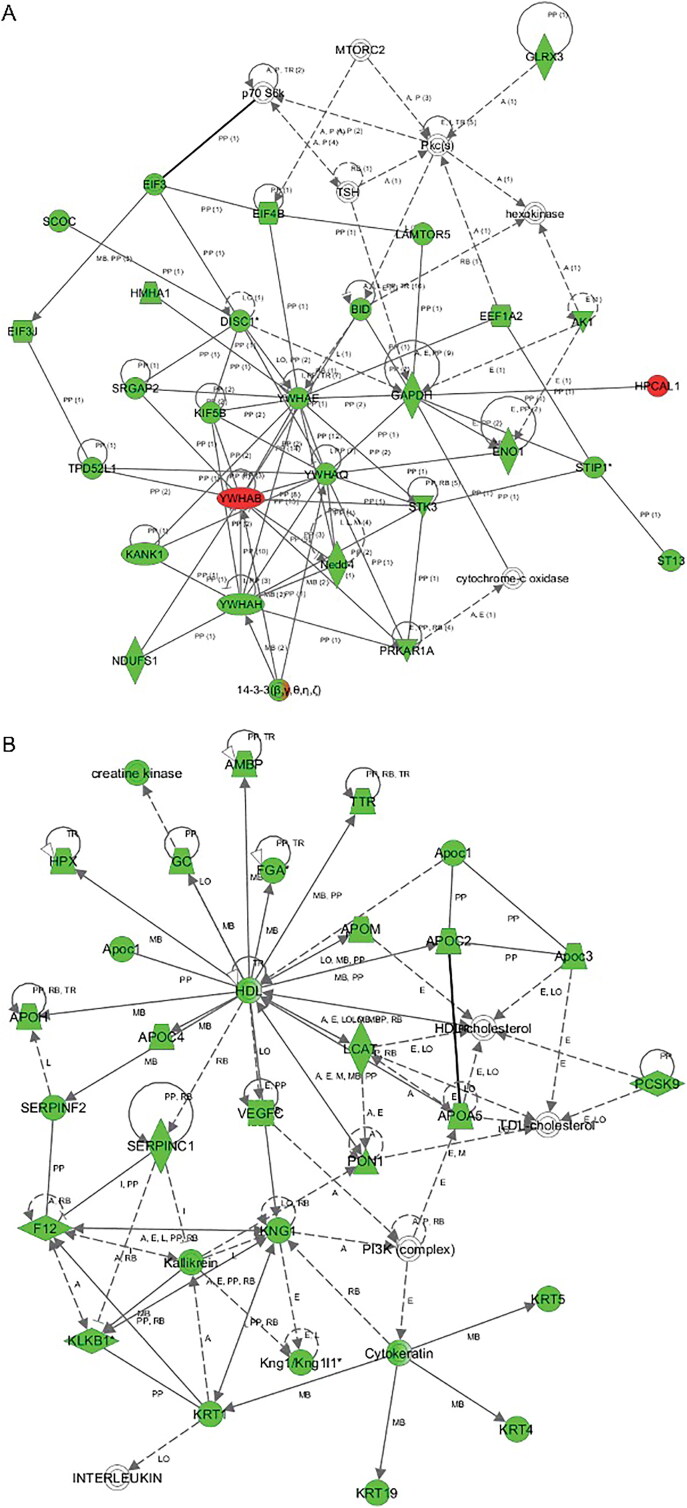
The major altered protein interaction networks after venom entrance in the low-dose (A) and high-dose (B) groups. Proteins in red and green represent upregulation and downregulation, respectively.

As shown in Figure 6, IPA network analysis of significant protein interactions also differed between the high- and low-dose groups. In the low-dose group, the three major networks were cell communication, cell organization and cell death. However, the high-dose group was mainly involved in metabolism disorder, protein synthesis blockade and molecular transport blockade.

### Dose-dependent effect of venom on serum proteins

In this study, two different venom dosages were applied: high-dose group (0.4 µg/g) and low-dose group (0.2 µg/g). At 1, 2 and 4 h post-venom injection, rat sera were collected, and proteome changes were compared over time. To further investigate the dose-dependent proteins, bioinformatics analysis was further performed on proteins with the same expression profiles; that is, their expression in the high-dose group should be consistently higher or lower than that in the low-dose group throughout the whole three time points. Furthermore, a 1.5-fold change was set as the threshold, and, thus, 11 significant proteins were obtained (Table 3).

**Table 3.** Dose-dependent significant proteins.

Trend	ID	Symbol	Entrez gene name	Type(s)
1	EGF_RAT	EGF	Epidermal growth factor	Growth factor
1	D4A6D9_RAT	HS1BP3	HCLS1 binding protein 3	Other
1	D3ZIA5_RAT	KIF18A	Kinesin family member 18A	Enzyme
1	D3ZPE8_RAT	SCARF1	Dcavenger receptor class F, member 1	Transmembrane receptor
1	S26A5_RAT	SLC26A5	Dolute carrier family 26 (anion exchanger), member 5	Transporter
1	G3V7N7_RAT	ZNF451	Zinc finger protein 451	Other
−1	BIN2_RAT	BIN2	Bridging integrator 2	Other
−1	HPT_RAT	HP	Haptoglobin	Peptidase
−1	Q5M8A0_RAT	KNG1	Kininogen 1	Other
−1	A1AG_RAT	ORM1	Orosomucoid 1	Other
−1	TAGL2_RAT	TAGLN2	Transgelin 2	Other

Trend 1 represents proteins that are highly expressed in the high-dose group and −1 represents proteins highly expressed in the low-dose group.

**Figure 7.**
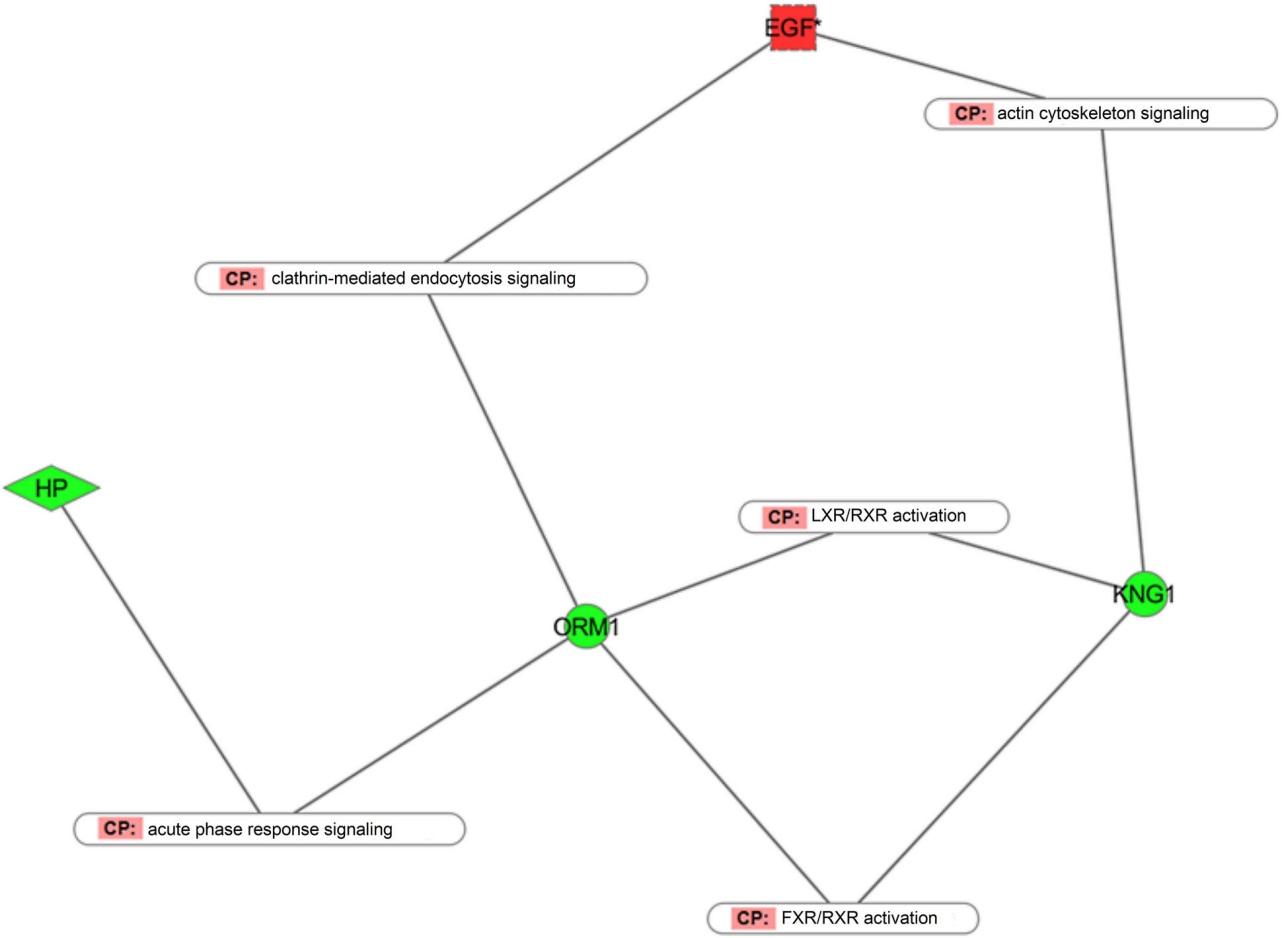
Pathways of the dose-dependent proteins. Ellipse icon shows the pathway names and the node colour displays the expression profile of the proteins. Red represents the increased expression of proteins with higher dose venom injection and green is the opposite.

Interestingly, all of the 11 significant proteins are relatively associated with cancer, which could also trigger the same host immune response as venom. In addition to cancer, these proteins are associated with some other biological functions, such as molecular transport, cell movement, cell adhesion and accumulation. Particularly, epidermal growth factor (EGF) is a growth factor that stimulates cell growth, proliferation, and differentiation by binding to its receptor EGFR. The dysregulation of EGF is associated with the development and progression of some cancers and responsible for hypomagnesemia [37,38]. Transmembrane protein SLC26A5 (solute carrier anion transporter family 26, member 5) is the motor protein of the outer hair cells of the mammalian cochlea. This protein could mediate the exchange of chloride across the plasma membrane [39], and its gene mutation could lead to sensorineural hearing loss [40]. BIN2 (bridging integrator 2) is associated with cell migration and invasion, and is involved in the formation of the pseudopod body and inhibition of phagocytosis [41]. Zinc finger protein 451 (ZNF 451) is a transcriptional cofactor that binds to specific DNA and RNA to modulate transcription. It is also the coactivator of the steroid receptor. TAGLN2 (transgelin-2) is one of the earliest markers of differentiated smooth muscle and is also a tumour growth inhibitor. Kininogen-1 (KNG1) is the precursor protein of high-molecular-weight kininogen (HMWK) and low-molecular-weight kininogen (LMWK), in which HMWK is essential for blood coagulation and assembly of the kallikrein–kinin system. Orosomucoid 1 (ORM1) is the serum acute phase protein and is involved in specific immune response. The pathways that these significant proteins are involved in are displayed in Figure 7.

Two metabolic pathways mediated by nuclear receptor, LXR\RXR activation and FXR\RXR activation, were both inhibited in the higher dose situation. LXR, forming a heterodimer with its partner RXR, is an important regulator of inflammation, fatty acid, cholesterol and bile acid metabolism. The FXR, in coordination with RXR, is involved in modulating lipoprotein, fatty acid and glucose metabolism via the regulation of bile acid levels. These results indicate that higher doses of venom could cause severe disorders of protein, cholesterol, fatty acid and glucose metabolism and thus resulted in death of rats 4 h post-venom injection.

Apart from the metabolism pathways, acute phase response, the host systematic defense response triggered by local inflammation was also inhibited when higher doses of venom were used. Acute phase response is a physiological process occurring soon after the onset of infection, trauma, tumour growth and immunological disorders, in which the plasma concentration of inflammatory cytokines and acute phase proteins is increased to maintain the host homeostasis. Such a defense response was activated in the early stage of snakebite, while it was inhibited over time or when higher doses of venom were injected.

Clathrin-mediated endocytosis signalling is used for the trafficking of nutrients, hormones and other signalling molecules from the plasma to intracellular regions. Actin cytoskeleton signalling is essential in some dynamic processes, such as cell movement, axon guidance cytokinesis and phagocytosis. The disorder of these two pathways indicated that higher dose venom would cause more severe damage to host material transmission and motor ability.

**Figure 8.**
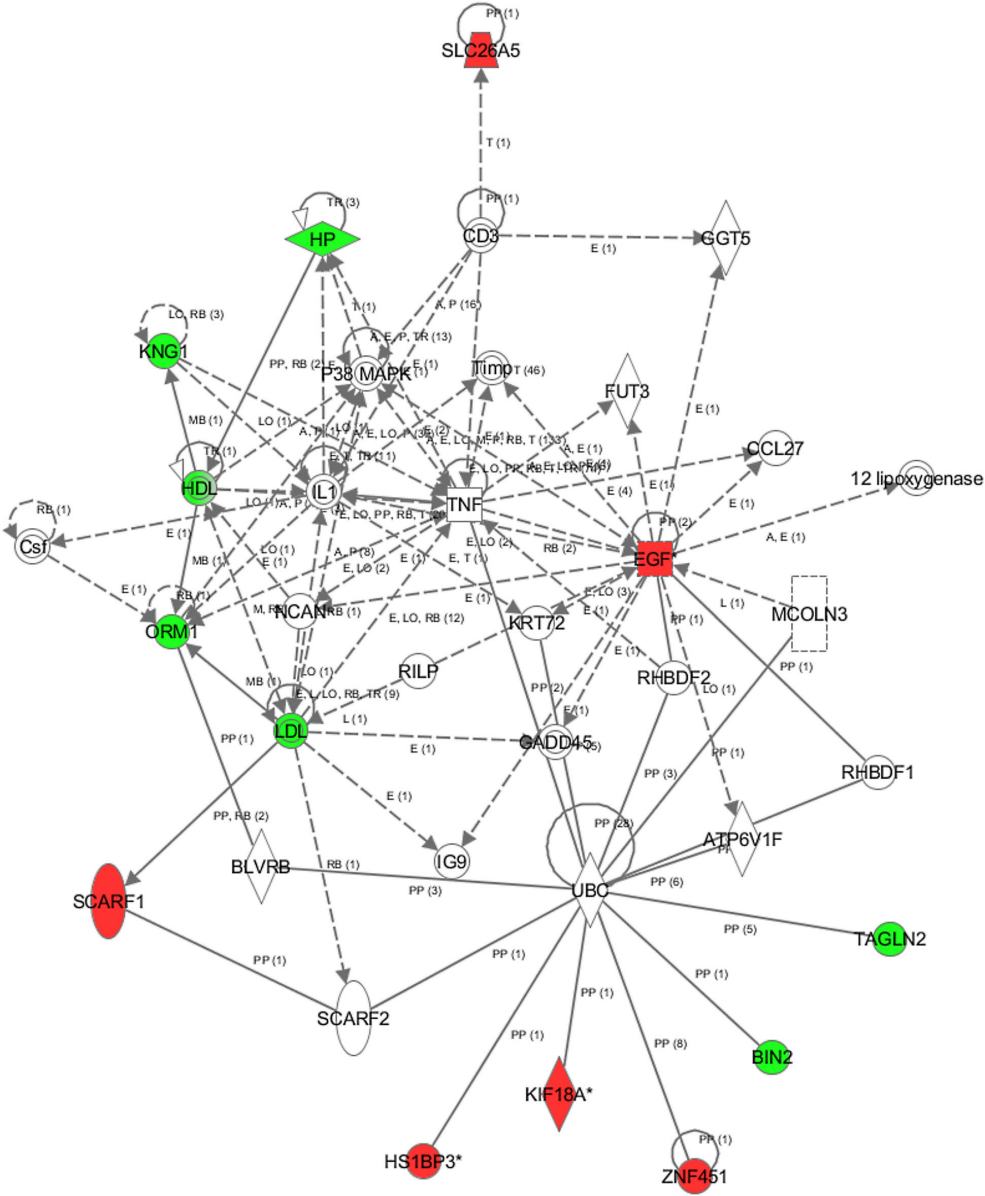
Networks of significant proteins. Red and green represent proteins that are upregulated and downregulated, respectively.

The networks of significant proteins are primarily associated with lipid and small molecular metabolism (Figure 8), in which KNG1 and ORM1, the proteins involved in metabolism and immune response, occupied a core position. As a result, these two proteins could serve as venom dose-dependent biomarker candidates.

## Conclusion

This study, for the first time, systematically investigated the effect of Chinese cobra snakebite time and venom dose on serum proteins using proteomics. We found that serum proteomes differed between different dosages of snake venom. Chinese cobra venom contains complex zootoxins, including haemotoxin and neurotoxin. In our study, there were 47 and 28 significantly changed proteins in the high-dose group and low-dose group, respectively. Some of these proteins were persistently upregulated or downregulated over the time course and could be the first choice for Chinese cobra snakebite identification. KNG1 and ORM1 were probably the potential biomarkers for the intoxication of Chinese cobra venom.
